# Hetero- and adaptive resistance to polymyxin B in OXA-23-producing carbapenem-resistant *Acinetobacter baumannii* isolates

**DOI:** 10.1186/1476-0711-12-15

**Published:** 2013-07-02

**Authors:** Juliana Barin, Andreza Francisco Martins, Bianca Lucia Heineck, Afonso Luis Barth, Alexandre Prehn Zavascki

**Affiliations:** 1 Post-Graduate Medical Sciences Program, Universidade Federal do Rio Grande do Sul, Porto Alegre, Brazil; 2 Biomedicine School, IPA, Porto Alegre, Brazil; 3 School of Biology, Universidade Federal do Rio Grande do Sul, Porto Alegre, Brazil; 4 Clinical Pathology Service, Hospital de Clínicas de Porto Alegre, Porto Alegre, Brazil; 5 Infectious Diseases Service, Hospital de Clínicas de Porto Alegre, 2350 Ramiro Barcelos St, Porto Alegre 90.035-903, Brazil

**Keywords:** *Acinetobacter baumannii*, Polymyxins, Polymyxin B, Colistin, Hetero-resistance, Adaptive resistance, Multidrug-resistance, Carbapenemase

## Abstract

**Background:**

Resistance rates to polymyxin B in surveillance studies have been very low despite its increasing use worldwide as the last resort therapy for multidrug-resistant Gram-negative bacilli. However, two other resistance phenotypes, hetero- and adaptive resistance, have been reported to polymyxin. We aimed to investigate the presence of polymyxin B hetero- and adaptive resistance and evaluate its stability in carbapenem-resistant *Acinetobacter baumannii* (CRAB) clinical isolates.

**Methods:**

CRAB isolates were recovered from hospitalized patients at three Brazilian hospitals. Hetero-resistance was determined by population analysis profile (PAP). Adaptive resistance was evaluated after serial daily passages of isolates in Luria-Bertani broth containing increasing polymyxin B concentrations. MICs of polymyxin B of colonies growing at the highest polymyxin B concentration were further determined after daily sub-cultured in antibiotic-free medium and after storage at −80°C, in some selected isolates.

**Results:**

Eighty OXA-23-producing CRAB isolates were typed resulting in 15 distinct clones. Twenty-nine randomly selected isolates (at least one from each clone) were selected for hetero- resistance evaluation: 26 (90%) presented growth of subpopulations with higher polymyxin B MIC than the original one in PAP. No isolate has grown at polymyxin B concentrations higher than 2 mg/L. Polymyxin B MICs of subpopulations remained higher than the original population after daily passages on antibiotic-free medium but returned to the same or similar levels after storage. Twenty-two of the 29 isolates (at least one from each clone) were evaluated for adaptive resistance: 12 (55%) presented growth in plates containing 64 mg/L of polymyxin B. Polymyxin B MICs decreased after daily passages on antibiotic-free medium and returned to the same levels after storage.

**Conclusions:**

The presence of subpopulations with higher polymyxin B MIC was extremely common and high-level adaptive resistance was very frequent in CRAB isolates.

## Background

The increasing worldwide prevalence of multi-drug resistant, *Acinetobacter baumannii*, a major nosocomial pathogen, particularly carbapenem-resistant strains, is of great concern, since treatment becomes restricted to very few options [1]. Polymyxins, both B and E (colistin), are “old” polypeptide antibiotics that re-emerged in clinical practice as the last resort therapy against multidrug-resistant Gram-negative bacteria; many, including *A. baumannii*, are only susceptible to these drugs [2]. Although resistance rates to polymyxins in surveillance studies fortunately remain very low [3], two relatively poorly understood resistance phenotypes, hetero- and adaptive resistance, have been reported in these drugs [4,5].

The term hetero-resistance refers to a phenotype characterized by the presence of different (drug-resistant and –susceptible) populations in a single clinical specimen or isolate [6], while adaptive resistance describes an autoregulated phenomenon characterized by rapid induction of resistance in the presence of drug and reversal to the susceptible phenotype in its absence [7].

Hetero-resistance has been recently described for colistin in some carbapenem-resistant *A. baumannii, Pseudomonas aeruginosa* and *Klebsiella pneumoniae* isolates [8-10], and other studies have demonstrated the presence of adaptive resistance to polymyxins, mainly in *P. aeruginosa*[7]. No study so far has neither investigated the presence of hetero-resistance for polymyxin B, the frequency of adaptive resistance in carbapenem-resistant *A. baumannii* (CRAB) isolates, nor assessed the presence of these distinct phenomena in the same isolates. The aim of this study was to assess the occurrence of these phenomena and evaluate its stability in CRAB clinical isolates.

## Methods

### Bacterial strains

CRAB isolates were selected from a total of 132 *Acinetobacter* spp. isolates (one isolate by patient) consecutively recovered from patients admitted to three tertiary-care hospitals from Porto Alegre, Brazil, from March to December 2011. Isolates were identified by Vitek 2 system. The following carbapenemase-encoding genes were examined by multiplex PCR: *bla*_OXA-23_, *bla*_OXA-24_, *bla*_OXA-51_, *bla*_OXA-58_ and *bl*a_OXA-143_ genes [11]. *A. baumannii* species was confirmed by the presence of the intrinsic *bla*_OXA-51_ gene [12].

MICs for polymyxin B, imipenem and meropenem were determined by broth microdilution and interpreted according to CLSI guidelines [13]. *Pseudomonas aeruginosa* ATCC 27853 was included as quality control in all tests.

CRAB isolates were submitted to molecular typing by *Apa*I DNA macrorestriction followed by PFGE [14]. Results were interpreted using a dendrogram constructed using the band-based Dice coefficient method, and, for the purpose of this study, isolates with >90% were considered a clone.

### Hetero-resistance

Hetero-resistance in selected CRAB isolates was determined by population analysis profile (PAP). Briefly, solutions containing seven distinct bacterial inoculum, ranging from 10^8^ (0.5 McFarland standard) to 10^2^ CFU/ml were prepared to facilitate bacterial counting in each plate. A 20 μL aliquot of each solution was spread on Mueller-Hinton agar plates containing 0, 0.5, 1, 2, 3, 4 and 6 mg/L of polymyxin B. Colonies were counted after 48 h of incubation at 35°C. The limit of counting was 20 CFU/ml. The frequency of hetero-resistant subpopulations at the highest drug concentration was calculated by dividing the number of colonies grown on antibiotic-containing plates by the colony counts from the same bacterial inoculum plated onto antibiotic-free plates [9]. MICs of polymyxin B of these subpopulations growing at the highest polymyxin B concentration were determined after daily sub-cultured in antibiotic-free medium for 4 days and after 75 days storage at −80°C, in some selected isolates.

### Adaptive resistance

Isolates were submitted to serial daily passages in freshly prepared Luria-Bertani (LB) broth containing increasing polymyxin B concentrations of 0.25 to 64 mg/L for a total of nine days (adapted from Fernandez *et al*. [15]. MICs of polymyxin B of colonies growing at the highest polymyxin B concentration were also determined after daily subculture in antibiotic-free medium for 3 days and after 60 days storage at −80°C, in some isolates.

## Results

Of the 132 *Acinetobacter* spp., 124 were confirmed as *A. baumannii* by the presence of *bla*_OXA-51_ gene, and 89 (71.7%) of these were CRAB isolates. All CRAB isolates were positive for *bla*_OXA-23_ and no product of amplification was detected for the other carbapenemase-encoding genes. Of these, 80 were typed, resulting in 15 distinct clones. MIC_50_ for both imipenem and meropenem were 64 mg/L and 32 mg/L and MIC_90_ were 128 mg/L and 64 mg/L, respectively. MIC of polymyxin B ranged from ≤0.125 mg/L to ≥64 mg/L. Twenty-nine randomly selected isolates (at least one from each clone) were selected for hetero-resistance evaluation.

PAP revealed the growth of subpopulation with 2- to at least 4 fold dilutions higher polymyxin B MIC than the original population in 26 (90%) of 29 isolates, including at least one isolate representative of each clone (Table 1). No isolate has grown at polymyxin B concentrations higher than 2 mg/L. The proportions of higher MIC subpopulations ranged from 2.5 × 10^-7^ to 6.2 × 10^-4^. MICs of polymyxin B of the 26 “higher MIC” subpopulations remained higher than the original population MIC after daily passages on polymyxin B-free medium (Table 1). After storage, MIC for polymyxin B among 17 selected subpopulations with higher MIC returned to levels similar to the original population, with most presenting exactly the same MIC of the original population.

**Table 1:** **Results of population analysis profile (PAP) of selected carbapenem-resistant *Acinetobacter baumannii *isolates**

**Strain**	**PFGE group**	**MIC (mg/L)**	**Highest concentration where growth occurred in population analysis (mg/L)**	**Frequency of appearance of subpopulations (PAP)**	**MIC after 4 days daily passages in drug-free medium (mg/L)**	**MIC after 75 days storage (mg/L)**
**1**	A	≤ 0.125	1	6.6 × 10^-5^	1	≤ 0.125
**2**	A	≤ 0.125	2	6 × 10^-5^	2	≤ 0.125
**3**	A	0.25	NG	NA	NA	NA
**4**	A	≤ 0.125	1	1 × 10^-7^	1	NP
**5**	A	0.25	1	5 × 10^-5^	1	NP
**6**	A	0.25	1	1.5 × 10^-6^	1	NP
**7**	B	≤ 0.125	0.5	5 × 10^-4^	0.5	≤ 0.125
**8**	B	0.25	1	2.5 × 10^-7^	1	0.25
**9**	B	≤ 0.125	0.5	1 × 10^-6^	0.5	NP
**10**	C	≤ 0.125	1	1.2 × 10^-5^	1	≤ 0.125
**11**	C	1	1	NA	1	1
**12**	C	≤ 0.125	1	7.1 × 10^-5^	1	NP
**13**	D	≤ 0.125	1	1.3 × 10^-6^	1	NP
**14**	D	≤ 0.125	1	3.3 × 10^-6^	1	NP
**15**	E	0.25	1	1 × 10^-6^	1	NP
**16**	F	0.5	2	8.3 × 10^-5^	2	1
**17**	F	≤ 0.125	1	1 × 10^-6^	1	≤ 0.125
**18**	G	0.25	1	7.5 × 10^-5^	1	0.25
**19**	H	0.25	1	3.3 × 10^-4^	1	0.25
**20**	I	0.25	2	3.3 × 10^-5^	2	1
**21**	I	0.5	1	3 × 10^-6^	1	1
**22**	J	0.25	1	1.4 × 10^-4^	1	0.25
**23**	K	0.25	NG	NA	NA	NA
**24**	L	≤ 0.125	0.5	1.5 × 10^-6^	0.5	≤ 0.125
**25**	L	≤ 0.125	1	4 × 10^-4^	1	NP
**26**	M	≤ 0.125	1	6.2 × 10^-4^	1	≤ 0.125
**27**	N	0.5	2	6.6 × 10^-5^	2	1
**28**	N	≤ 0.125	0.5	1.5 × 10^-5^	0.5	NP
**29**	O	≤ 0.125	1	5 × 10^-5^	0.5	0.5

*PAPs* population analysis profiles, *NA* not applicable, *NG* no growth, *NP* not performed.

Twenty-two of the 29 isolate (at least one from each clone) were evaluated for adaptive resistance. In twelve isolates, growth was observed in plates containing 64 mg/L of polymyxin B (Table 2). After daily passages on polymyxin B-free medium for 3 days the MIC of isolates growing at 64 mg/L remained the same for two isolates and decreased 1- to 2-fold dilutions for the other ten (Table 2). Polymyxin B MICs after 60 day storage (performed in four isolates) were exactly the same of the MIC of baseline.

**Table 2:** **Results of adaptive resistant experiments of selected carbapenem-resistant *Acinetobacter baumannii *isolates**

**Strain**	**PFGE group**	**MIC (mg/L)**	**Polymyxin B MIC of subpopulations selected in PAP**	**Highest concentration of polymyxin B where growth was observed (mg/L)**	**MIC after 3 days daily passages in drug-free medium (mg/L)**	**MIC after 60 days storage (mg/L)**
2	A	≤ 0.125	2	64	32	NP
3	A	0.25	NA	64	16	NP
5	A	0.25	1	NG	NA	NA
6	A	1	1	64	16	NP
7	B	≤ 0.125	0.5	64	16	NP
8	B	0.25	1	64	16	NP
9	B	≤ 0.125	0.5	64	16	NP
10	C	≤ 0.125	1	64	32	≤ 0.125
12	C	≤ 0.125	1	64	16	NP
14	D	≤ 0.125	1	NG	NA	NA
15	E	0.25	1	NG	NA	NA
16	F	0.5	2	NG	NA	NA
17	F	≤ 0.125	1	NG	NA	NA
18	G	0.25	1	64	≥ 64	NP
19	H	0.25	1	NG	NA	NA
20	I	0.25	2	NG	NA	NA
22	J	0.25	1	64	16	0.25
23	K	0.25	NA	NG	NA	NA
24	L	≤ 0.125	0.5	64	16	≤ 0.125
26	M	≤ 0.125	1	64	≥ 64	≤ 0.125
27	N	0.5	2	NG	NA	NA
29	O	≤ 0.125	1	NG	NA	NA

PAP population analysis profiles, *NA* not applicable, *NG* no growth, *NP* not performed.

## Discussion

Our study for the first time investigated the presence of hetero- and adaptive resistance to polymyxin B in unrelated OXA-23-producing CRAB isolates. Additionally, the stability of these phenomena was evaluated in two distinct conditions. Since the susceptibility breakpoint for polymyxin B according to CLSI is 2 mg/L [13], real hetero-resistance for polymyxin B was not found in any isolate, differently from previous studies with colistin [9,16,17]. However, the presence of “higher MIC” subpopulations, within the susceptibility range, was detected in 90% of tested isolates, including at least one isolate representative of each of the 15 clones. These “higher MIC” subpopulations presented MICs 2- to at least 4-fold dilutions higher than the original population.

The presence of adaptive resistance to polymyxin B was shown in 55% of 22 tested isolates (present in 7 of 15 clones), all demonstrating high-level resistance to polymyxin B (MIC = 64 mg/L). Although some molecular mechanisms of adaptive resistance to polymyxins, such as mutations in *pmr*CAB and *lpxA* gene in *A. baumannii*[18,19] and PhoP-PhoQ, PmrA-PmrB and recently ParR-ParS in *P. aeruginosa*[15], have been characterized, the presence of this resistance phenotype has not been systematically evaluated. Thus, our study further suggests that adaptative resistance might be most common than possibly expected, at least in CRAB, since approximately half of tested clones showed such adaptive phenotype. Indeed, the frequency might be even higher if the agar plate with the lowest polymyxin B concentration had <0.25 mg/L of the drug. Although seven isolates with MIC ≤0.125 mg/L still have growth on these plates, these concentrations may have inhibited the growth of other eight isolates. 

The present study also showed that the MIC of the “higher MIC” subpopulations remained stable after 4-days into antimicrobial-free medium, but returns to the MIC of the original population after storage at −80°C, suggesting that it might involve some molecular basis also associated with an unstable phenotype. As expected, since without the drug-sustaining effect the adaptive resistance is unstable, the MICs of resistant isolates selected in the adaptive resistance experiment decreased 1- to 2-fold dilutions after serial passage into antimicrobial-free medium and all tested isolates returns to the baseline level after the storage at −80°C.

Only one isolate that has presented adaptive resistance has not presented “higher MIC” subpopulation in PAP. It belongs to the clone A, which has other three isolates tested in both experiments, all showing the presence of both phenomena. It is also interesting that these latter three isolates were identical by typing while the former showed 92% of similarity with these latter ones (data not shown). Another isolate has neither presented “higher MIC” subpopulation nor adaptive resistance and belongs to a clone with two representative isolates among the 80 CRAB typed in this study.

Unfortunately, we were not able to determine the molecular determinants of these phenotypes in this study. We also could not determine if the absence of real hetero-resistance (i.e. presence of subpopulations with MICs higher than the susceptibility breakpoint) was a specific characteristic of polymyxin B, and would occur with colistin, or “higher MIC” subpopulations within the susceptibility range was only detected, instead of subpopulations with “resistance MICs” because the baseline MIC of half of the tested isolates were very low (≤0.125 mg/L).

In summary, our study showed that the presence of “higher MIC” subpopulations in CRAB isolates was extremely common. Additionally, high-level adaptive resistance was also very frequent. The clinical significance of each phenomenon should be further investigated, since both may potentially affect the outcomes of patients on therapy with polymyxins.

## Competing interests

APZ has received consultancy fees from Pfizer, Eurofarma and Forest Laboratories. All other authors: none to declare.

## Authors’ contributions

JB was responsible for performance of the experiments, data interpretation and drafting the manuscript; BLH performed the experiments and contributed to manuscript draft; AFM and ALB contributed in the experiments, data interpretation and manuscript draft; and APZ conceived the study, contributed in data interpretation, drafting and reviewing of the manuscript. All authors read and approved the final manuscript.
